# Insights on the science of team science: 15 years and counting

**DOI:** 10.1017/cts.2025.10134

**Published:** 2025-09-02

**Authors:** Colleen Cuddy, Madison L. Hartstein, Whitney Sweeney

**Affiliations:** 1 Lane Medical Library, Stanford University School of Medicine, Stanford, CA, USA; 2 Northwestern University Clinical and Translational Sciences Institute (NUCATS), Feinberg School of Medicine, Chicago, IL, USA; 3 Institute for Clinical and Translational Research, School of Medicine and Public Health, University of Wisconsin-Madison, Madison, WI, USA

**Keywords:** Team science, science of Team Science, artificial intelligence, translational science, interdisciplinary research

## Abstract

The fifteenth annual Science of Team Science Conference, “Insights on the Science of Team Science: 15 Years and Counting,” was held virtually from July 30 to August 1, 2024. Two hundred participants from diverse backgrounds and sectors celebrated the evolution of team science and looked to the future. This paper presents a summary of the conference proceedings, highlighting keynotes, workshops, presentations, and posters that explored innovations in the science of team science. Notable topics included the integration of artificial intelligence (AI) in teams and methods for evaluating team effectiveness. The conference fostered networking opportunities through an interactive virtual platform, enhancing community building among attendees.

## Introduction

Science of Team Science (SciTS) as a field has grown exponentially since its introduction at an inaugural conference in 2010. SciTS continues to encompass both conceptual and methodological strategies aimed at understanding and enhancing the processes and outcomes of collaborative, team-based research [1]. In a world where societal and scientific grand challenges increasingly require a cross-disciplinary collaborative approach, SciTS research has produced a large body of literature. Current SciTS research questions include

- How do we use team theory (e.g., managing and training teams) to help scientific teams?
- How does studying communication in science teams help us understand scientific collaboration?
- How do scientists collaboratively solve problems?
- How does leading science teams differ from leadership in other areas?

SciTS explores practical applications to facilitate effective teamwork and better evaluate team science using large-scale studies involving bibliometrics to more proximal measures such as subjective assessments of team processes, including communication, collaborative problem-solving, affect, and leadership [2].

The International Network for the Science of Team Science’s (INSciTS) annual conference has established itself as the premier annual gathering of scholars and practitioners in the SciTS. Through a combination of workshops, panels, presentations, posters, and intentional networking opportunities, a global community of researchers, administrators, students, funders, and policymakers from academia, government, and industry shared their expertise. Held virtually from July 30 to August 1, 2024, “Insights on the Science of Team Science: 15 Years and Counting” (SciTS2024) connected over 200 attendees by looking to the future while celebrating the conference’s fifteenth anniversary. Iterations and innovations in team effectiveness and newer research areas with practical and theoretical implications for the SciTS, such as the use of artificial intelligence (AI) in team science, resilience and adaptability in teams, and big team science, were woven throughout the conference program. Highlights of the program are discussed below.

### Keynote sessions

Three keynotes anchored the conference. In an opening conversation, facilitator Maritza Salazar Campo (University of California-Irvine) interviewed keynote speakers Kara Hall (National Cancer Institute) and Daniel Stokols (University of California-Irvine). As co-organizers of the first SciTS conference [1], these visionaries offered the audience a reflection on the state of the field as it moved from its early focus on expanding the limits of singular approaches to science by defining conceptual models to developing more nuanced social models of intellectual synergy and integration. The session concluded by encouraging scholars in SciTS to continue to be curious, find connections across disciplines, and embrace different methods and approaches. The speakers cautioned against devaluing differing approaches, emphasizing that structural views of teaming and microprocesses all contribute to the fabric of the SciTS. Hall and Stokols appealed to the audience to develop leaders and scholars who can utilize a pluralistic lens of evaluation to see promise outside of narrow lanes and bridge multiple levels of the scientific enterprise. The discussion ended with a closing precaution to be wary of geopolitical and narrow nativistic sentiments that may impact inclusionary practices and the study of interdisciplinary science.

**Figure 1.**
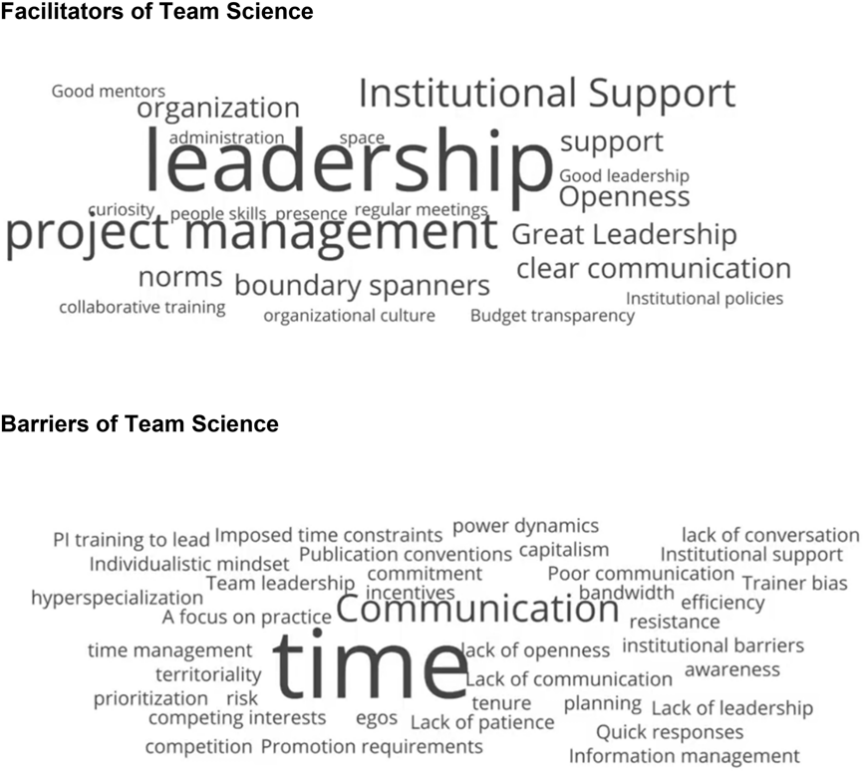
Team science barriers and facilitators. These word clouds were generated from polls of the audience conducted by Maritza Campo Salazar during the opening keynote. The top panel shows the facilitators of team science, and the bottom panel shows the barriers of team science based on audience input to the polls.

During the keynote, Salazar Campo polled the audience on what they experienced as team science facilitators and barriers, creating thematic word clouds. Leadership and institutional support were examples of standout facilitators of team science, while (lack of) time and communication were salient barriers. These themes reverberated throughout the conference Figure 1 shares the outcome of the polls in word clouds.

Demonstrating the value and impact of effective multidisciplinary teams on real-world outcomes is challenging but necessary to move the field forward. To address this important topic, Chris Wiese (Georgia Institute of Technology) moderated a discussion on evaluating the effectiveness of SciTS with academic and practitioner experts representing a variety of fields from philosophy to public health to marine sciences. Dorothy Carter (Michigan State University), Katie Plaisance (University of Waterloo), Katia Noyes (University of Buffalo), LaKaija Johnson (Sage Bionetworks), and Sofia Ibarraran-Viniegra (Woods Hole Oceanographic Institution) shared their experiences on how to produce better scientific teams. The panel discussed various metrics for evaluating collaboration progress, including the frequency of new partnerships, the stages of team development, and the range of journals in which teams are publishing. Johnson also highlighted the Translational Science Benefits Model (TSBM) as a useful framework for fostering successful multidisciplinary team outcomes [3]. The panel further explored how maximizing efficiency, such as leveraging existing data, can expand publication reach and optimize the use of time and resources in these complex collaborations [4].

The conference concluded with experts Beth Burnside (University of Wisconsin-Madison), Christopher Lindsell (Duke University), Ashok Krishnamurthy (University of North Carolina), and Desiree Zercher (University of Mannheim) sharing their research in the rapidly evolving field of AI in the SciTS. The conversation highlighted themes such as large language models (LLMs), predictors of knowledge, skills, and attitudes (KSAs), and working with AI as a team member. Speakers shared their research as an entry to the broader social, ethical, and practical implications of AI in team science via a guided discussion led by Jim Spohrer (Retired–IBM, Cognitive OpenTech). The presentations invited discussion surrounding the ethics, advancements, hindrances, and potential that AI holds for the future of team science.

### Workshop highlights

Interactive workshops have been a key component of the conference since its inception. SciTS2024’s workshops shared developing knowledge for supporting team science and building community. The annual “Team Science 101” workshop led by INSciTS board members provided an overview of team science, covering its evolution, key terminology, and methodologies. It is a popular foundational session for those new or returning to the field to better understand SciTS’s scope and applications.

Themes of cultural competence, learning from failure, and stakeholder collaboration ran throughout the workshops. A continuing challenge for team science involves integrating cultural competencies into practice. Michelle Bennett’s (Roger Schwarz and Associates) workshop highlighted the need for awareness of cultural differences in team dynamics. Another timely topic was learning from failure. Whitney Sweeney’s (University of Wisconsin-Madison) “Failing to Succeed: Leveraging Failure to Improve and Innovate” presented a systems approach to analyzing team failures to learn, adapt, and innovate. Other workshops also highlighted the need for ongoing evaluation of team performance and dynamics, including exploring frameworks like the Internal Permissions Framework (IPF) and promoting a “debrief culture” to identify challenges and opportunities for growth within teams. Workshop attendees also learned how to strategically plan, organize, and conduct successful science gatherings; engage with participants; integrate feedback; and leverage tools like the Responsibility Assignment Matrix (RACI) and Objectives and Key Results (OKR).

### Oral presentations and research highlights

Speakers shared new and innovative research via thirty-one oral and five panel presentations. Presentations were organized by topics: leadership, knowledge integration, enhancing collaboration, engaging communities, case studies, and boundary management, and highlighted the impact of effective communication, inclusive practices, and dedicated support structures in advancing scientific knowledge and addressing complex challenges.

Specific topics included:

- “Novel methods for evaluating team success” (Bijana Birac and Karen DeMeester, University of Georgia);
- “Resilience and adaptation in large interdisciplinary teams” (Colleen Cuddy, Stanford University);
- ‘Encouraging citizen science with the Team Science Community Toolkit’ (Madison L. Hartstein, Northwestern University);
- “Advancing international research collaborations” (Takehito Kamata, Sophia University) and (Santo Fortunato, Indiana University; Christine Hedren, Appalachian State University; TJ Ronnigen, The Ohio State University; Leslie Smith, Your Ocean Consulting, LLC & Deep Ocean Observing Strategy; Marisa Rinkus, Michigan State University);
- “Enhancing knowledge integration and collaboration through reflexivity” (David Fuentes, University of Portland, and Jeremy Hughes, Chicago State University);
- “Advancing innovative health solutions with effective project management” (Whitney Sweeney, University of Wisconsin-Madison).

Camille Santistevan and co-authors from the Center for Scientific Collaboration and Community Engagement won the SciTS2024 Best Paper Award for their paper entitled “Facilitating Collaboration and Connection: How an 8-Week Online Training is Improving Community Building in STEM.” Their research, based on a study of over 300 participants, described how a training course designed for community managers provided actionable strategies and frameworks to enhance roles and foster professional connections.

### Poster presentations

Three main themes emerged from the poster session: collaboration, training and education, and methodological innovations. “Collaboration” posters focused on the dynamics and processes of team science, including the development of team science skills, the structure of international virtual research organizations (IVROs), and the importance of effective communication and coordination within research teams.

“Training and education” posters described evidence-based training (TeamMAPPS) and the application of adult learning principles (andragogy) to design effective training for scientists. “Innovations” posters included science gateways used to facilitate access to research infrastructure, data, and tools, promoting open science and the exploration of electronic systems with data repositories to enhance coordination and communication within distributed work teams.

The SciTS2024 Best Poster Award was presented to Kimberly Bourne (Appalachian State University) and Alison Deviney (North Carolina State University) for their poster, “Knowledge Mapping in a Multidisciplinary Convergence Research Center.” Their work focused on innovative techniques for visualizing and integrating knowledge across diverse research teams to tackle complex societal challenges.

SciTS2024 introduced a new People’s Choice Award for Best Poster. Elizabeth Bello and co-authors from the University of Illinois at Urbana–Champaign received the award for their poster, “A Structured Review Tests the Value of Transdisciplinarity in Bioinspired Design (BID) Research” which examined the extent that BID research is transdisciplinary and how the makeup of a co-authorship team influences the content of the products they produce.

### Networking and community building in a virtual environment

**Figure 2.**
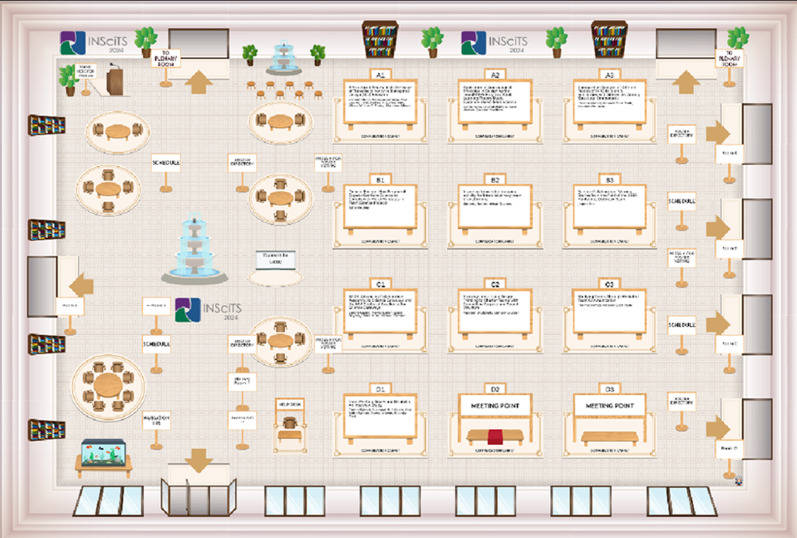
Lobby in the virtual conference environment. This screenshot shows the conference lobby as created by “Virtual chair,” powered by GatherTown. N.B. the setup for poster presentations and areas available for both planned meetings and impromptu conversations.

The value of networking and building community at conferences has been well documented as a critical component of team building and advancing scientific teamwork [5]. Even in a virtual setting, this conference was designed with an emphasis on creating opportunities for attendees to connect, engage, and build lasting relationships. The virtual platform “Virtual Chair,” powered by GatherTown, allowed attendees to get close to a “real-world” experience. Interactive opportunities to customize and tailor the conference experience allowed attendees to design a custom avatar, have private conversations with other attendees, and “walk around” to meet new people from around the world. This was particularly effective for the poster sessions. Figure 2 shows the virtual poster session. By facilitating connections, the conference supported current research and also helped to lay the foundation for future collaborative projects. Building a strong community within team science is also a core tenet of the mission of INSciTS and is essential to furthering research and practice.

### Key takeaways and resources from SciTS2024

SciTS2024 offered a dynamic opportunity to advance our understanding of scientific teamwork, focusing on the evolution and future direction of the science of team science. The conference provided valuable insights into effective collaboration and highlighted key trends in the field. Key takeaways from the conference’s development and the discussions include:

- **Innovative methods for virtual conferences**: Exploring best practices for creating engaging and interactive virtual events while maximizing the potential of real-world simulated networking opportunities.
- **Metrics for evaluation, collaboration, and team effectiveness**: Understanding the tools and metrics necessary for assessing the progress of collaborative efforts and evaluating team effectiveness in the context of team science.
- **Sharing resources and tools**: Disseminating key resources that foster the advancement of science through the Science of Team Science.

An abbreviated list of resources shared during the conference includes:

- Translational Science Benefits Model (TSMB)
- Responsibility Assignment Matrix (RACI)
- Objectives and Key Results (OKR)
- Team Science Community Toolkit (TSCT)
- Internal Permissions Framework (IPF)
- Publications about science gateways and innovative methodologies

These resources contribute to strengthening the infrastructure of collaborative research and promote the continued progress of the SciTS.

## Conclusion

SciTS2024 marked a significant milestone in the evolution of the field, celebrating fifteen years of progress while simultaneously charting a course for the future. It contributed to the application and advancement of the SciTS and showcased the multifaceted nature of the discipline. The ongoing exploration of methodologies, competencies, and strategies showcased remains crucial for enhancing the effectiveness of interdisciplinary research teams.

The integration of emerging technologies, particularly AI, within team science underscores the importance of adapting to new ways of working and anticipating future trends that can elevate interdisciplinary research practices. The conference’s content emphasized not just the value of teams in scientific inquiry but also the ethical and practical implications of leveraging technology for team dynamics and the importance of funder relationships and community engagement. The effective integration of the Virtual Chair conference platform underscored the SciTS community’s commitment to leveraging technology for enhanced collaboration.

Looking ahead, it is imperative that the insights and connections gained during this conference are employed to further advance the SciTS. The opening call to cultivate a pluralistic approach to evaluation and practice is critical to moving the field forward, as is the call for inclusivity in our community and our research. It will be increasingly important for the SciTS community to remain agile in the face of rapid technological advancements. These developments will reshape the way we collaborate, innovate, and address complex challenges. Through engagement, reflection, and curiosity, the SciTS community will continue to drive the evolution of team science and transform research practices, strengthening our collective capacity to address future challenges and, ultimately, transform scientific research for the better.

## References

[1] Falk-Krzesinski HJ , Börner K , Contractor N , et al. Advancing the science of team science. Clin Transl Sci. 2010;3:263–266. doi: 10.1111/j.1752-8062.2010.00223.x. 20973925 PMC2965626

[2] Brasier AR , Burnside ES , Rolland B. Competencies supporting high-performance translational teams : a review of the sciTS evidence base. 2023;3:e62. doi: 10.1017/cts.2023.17. PMC10052558 37008597

[3] Luke DA , Sarli CC , Suiter AM , et al. The translational science benefits model: a new framework for assessing the health and societal benefits of clinical and translational sciences. Clin Transl Sci. 2018;11:77–84. doi: 10.1111/cts.12495. 28887873 PMC5759746

[4] Bengert E , Towle-Miller L , Boccardo J , et al. Novel approach for tracking interdisciplinary research productivity using institutional databases. J Clin Transl Sci. 2022;6:e119. doi: 10.1017/cts.2022.455. 36259067 PMC9550599

[5] McClements DJ , McClements J , McClements IF. Conferences and networking: getting to know the science and the people behind it, In How to be Successful Scientist. Switzerland: Springer Nature; 2024:173–192. doi: 10.1007/978-3-031-51402-9_8.

